# The role of fluorine-18-fluorodeoxyglucose positron emission tomography in evaluating the response to tyrosine-kinase inhibitors in patients with metastatic primary renal cell carcinoma

**DOI:** 10.2478/raon-2013-0067

**Published:** 2014-07-10

**Authors:** Carmelo Caldarella, Barbara Muoio, Maria Antonietta Isgrò, Emilio Porfiri, Giorgio Treglia, Luca Giovanella

**Affiliations:** 1 Institute of Nuclear Medicine, PET/CT Center, Catholic University, Rome, Italy; 2 School of Medicine, Catholic University, Rome, Italy; 3 Institute of Biochemistry and Clinical Biochemistry, Catholic University, Rome, Italy; 4 School of Cancer Sciences, University of Birmingham, Birmingham, United Kingdom; 5 Department of Nuclear Medicine and PET/CT Center, Oncology Institute of Southern Switzerland, Bellinzona, Switzerland

**Keywords:** fluorodeoxyglucose, positron emission tomography, advanced renal cell carcinoma, tyrosine-kinase inhibitors, response to treatment

## Abstract

**Background:**

Positron emission tomography-computed tomography (PET-CT) using fluorodeoxyglucose (FDG) is increasingly used in the evaluation of patients with advanced renal cell carcinoma (RCC), primarily for staging purposes. The aim of this paper is to perform a systematic review about the usefulness of PET-CT using FDG in response assessment after treatment with tyrosine-kinase inhibitors (TKIs) in patients with advanced RCC.

**Materials and methods.:**

The scientific literature about the role of PET-CT using FDG in the assessment of response to treatment with TKIs in patients affected by advanced RCC was systematically reviewed.

**Results:**

Seven studies about the role of PET-CT using FDG in the response assessment after treatment with TKIs (essentially sunitinib and sorafenib) in advanced RCC were retrieved in full-text and analysed, to determine the predictive role of this morpho-functional imaging method on patient outcome.

**Conclusions:**

To date, the role of PET-CT using FDG in evaluating the response to TKIs in metastatic RCC patients is still not well defined, partly due to heterogeneity of available studies; however, PET-CT reveals potential role for the selection of patients undergoing therapy with TKIs. The use of contrast-enhanced PET-CT appears to be promising for a “multi-dimensional” evaluation of treatment response in these patients.

## Introduction

Primary renal malignancies are relatively uncommon tumours which can arise from either renal cortex or transitional epithelium of intra-renal urinary tract and pelvis. Renal cell carcinoma (RCC) accounts for about 3% of all adult malignant neoplasms in the United States (the 14^th^ most common malignancy worldwide in 2002), with an estimated incidence rate of 0.7–1.5 cases per 100 000 persons per year; renal pelvis malignancies are less common, with an estimated incidence rate of about 0.5 cases per 100 000 persons per year.^1^,^2^ Several risk factors, such as cigarette smoking, obesity, hypertension, diabetes mellitus and reproductive factors, besides genetic predisposition (von Hippel-Lindau disease), have been identified for RCC, which may explain the high variability of incidence rates worldwide.^3^,^4^ Clear cell carcinoma is the most common histological subtype of RCC (82%), followed by papillary carcinoma (11%); chromophobe, collecting duct carcinoma and unclassified RCC are far less common.^5^

Early detection of the primary tumour is desired, but disease–related symptoms (like haematuria, flank pain, fever and weight loss) are non-specific, therefore about 20–30% of patients have metastatic disease at diagnosis, with an expected 5-year survival of approximately 10%. Microscopic metastatic disease may also be apparent several years after curative nephrectomy.^6^–^8^ Patients with metastatic RCC are usually resistant to conventional cytotoxic chemotherapy agents and radiation therapy; efficacy of interleukin-2 and interferon alpha is limited, with an important toxicity burden. However, in a single centre experience, Motzer *et al*. have reported a significant rise in 2-year-survival rate from 3% (without therapy) to 20% (after interleukin-2, interferon alpha or both) in 670 patients with metastatic RCC.^9^ Selective multi target receptor tyrosine-kinase inhibitors (TKIs), like sunitinib and sorafenib, have been recently approved as novel therapeutic antiangiogenetic agents for treatment of advanced RCC, with reported satisfactory results on progression-free survival and quality of life.^10^–^13^

Positron emission tomography – computed tomography (PET-CT) is a combined functional and morphological nuclear medicine imaging technique which uses radio-labelled substances (radiopharmaceuticals) to visualize particular metabolic characteristics of either normal or pathological tissues. Fluorine-18-fluorodeoxyglucose (FDG) is a radioactive analogue of glucose, which is intravenously injected to detect increased glycolytic activity in tumour tissues. Unlike most cancers which show intense accumulation of FDG, due to their high glucose metabolism, RCC shows variable intensity of FDG uptake; besides, physiological urinary excretion of FDG makes it difficult to assess the metabolic activity of the primary tumour.^14^ However, PET-CT using FDG performs better in the detection of distant metastases, with sensitivity and specificity values of 79% and 90%, respectively, as reported on a recent meta-analysis, but with poorer performance on the detection of the primary neoplasm.^15^,^16^

More recently, PET-CT using FDG has been used more and more extensively to assess the treatment response to TKIs in patients with metastatic RCC; however, to date, a systematic evaluation of these studies does not exist in the literature. Therefore, the aim of our paper is to systematically and critically review the published data on this setting to assess the role of PET-CT using FDG in evaluating the treatment response to TKIs in patients with metastatic RCC.

## Materials and methods

### Search strategy

A comprehensive computer literature search of PubMed/MEDLINE, Scopus, and Embase databases was carried out to find relevant published articles concerning the evaluation of treatment response in patients with metastatic RCC undergoing therapy with TKIs. We used a search algorithm based on a combination of the terms: (‘‘PET’’ OR ‘‘positron emission tomography’’) AND (‘”kidney” OR “renal”). No language restriction was used. The search was performed from inception to October 17^th^, 2012. To expand our search, references of the retrieved articles were also screened for additional studies.

### Study selection

Studies or subsets in studies investigating the role of PET-CT using FDG in patients with metastatic RCC undergoing therapy with TKIs were eligible for inclusion. Case reports, small case series, review articles, letters, editorials and conference proceedings were excluded. The following inclusion criteria were applied to select studies for this systematic review:

PET-CT using FDG performed in patients undergoing therapy with TKIs for metastatic RCC,

A sample size of at least 5 patients with meta-static RCC who underwent PET-CT using FDG after treatment with TKIs, with available data about baseline pre-treatment PET-CT,

Available follow-up data about patient outcome, like progression-free survival (PFS) and/or overall survival (OS),

No data overlap (when possible duplicate studies were found; only the most complete article was included).

Two researchers (CC and GT) independently reviewed the title and abstract of the retrieved articles, applying the above-mentioned selection criteria. Articles were rejected if clearly ineligible. The same two researchers then independently evaluated the full-text version of the included articles to determine their eligibility for inclusion.

### Data extraction

Information about basic study data (authors, journal, year of publication, country of origin), study design (prospective or retrospective), patient characteristics (number of patients with metastatic RCC with PET-CT evaluation after therapy) and outcome data (PFS, OS) were collected. Only studies providing such complete information were included.

### Quality assessment

Two independent reviewers evaluated the methodology of the selected studies using the Quality Assessment Tool for Diagnostic Accuracy Studies (QUADAS).^17^ This 14-items tool is composed by five items related to verification bias, three items related to review bias, two items relating to generalizability and context and spectrum bias, and four to reporting. Reviewers, who were blinded to the purposes of the meta-analysis, recorded a score of “1” for “yes” and “0” for “no” for each of the 14 items; all disagreements were resolved by means of consensus. Inter-rater reliability was also evaluated.

## Results

### Literature search

**FIGURE 1.**
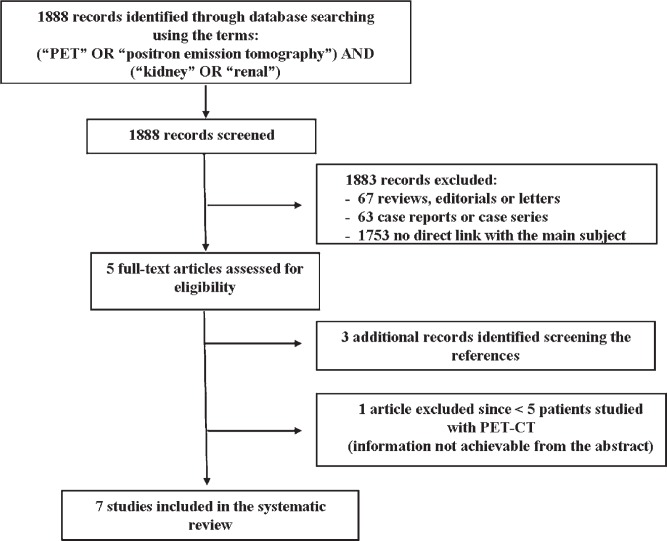
Literature search.

The comprehensive computer literature search from PubMed/MEDLINE, Embase and Scopus databases revealed 1888 articles (Figure 1). Reviewing titles and abstracts, 5 articles were potentially eligible for inclusion applying the selection criteria mentioned above and were retrieved in full-text version.^18^–^22^ Three additional studies were retrieved screening the references.^23^–^25^ One study was ultimately rejected since the full-text version analysis revealed that only one patient underwent PET-CT for evaluation of response to therapy, while baseline study was performed in all subjects.^21^ Most papers were excluded because they were not related to the main subject of this review.

Seven studies, comprising a total sample size of 137 patients with metastatic RCC, met all inclusion criteria and were included in this systematic review. The characteristics of these studies are summarized in Tables 1–3.

### Quality assessment

Overall, the seven studies included in this systematic review have shown moderate methodological quality according to QUADAS. Studies scored between 7/14 and 11/14 with a median score of 9/14. The index test and the reference standard were often interpreted without blinding, and this represents the most critical issue about the methodological quality of the included studies.

### Literature analysis

A preliminary prospective study about the usefulness of PET-CT using FDG in evaluating the early metabolic response to therapy with TKIs in patients with metastatic RCC was conducted by Vercellino *et al*.^22^ Twelve patients (overall 29 metastatic sites) were assessed with PET-CT at baseline and after the first cycle of sunitinib therapy was completed (at day 42): the metabolic response was assessed using European Organization for Research and Treatment of Cancer (EORTC) criteria, while CT (performed at day 84) was used to evaluate the morphological response according to Response Evaluation Criteria in Solid Tumors (RECIST).^26^,^27^ Maximum Standardized Uptake Value (SUVmax) and percentage variation in SUVmax (%SUVmax) were considered on a lesion-based analysis to assess the entity of response to the treatment. Patients with metabolic partial response on post-therapy PET-CT showed long PFS, but metabolic status was not predictive of clinical status at last follow-up: as the authors themselves stated, results were not statistically significant because of the small sample size. Increase of lesion size on CT was not predictive of progression: despite overall %SUVmax of −17%, the sum of lesion size remained unchanged. Particularly, in 2 patients with non-significant SUVmax decrease, a slight (non-significant) increase in lesion size, associated to reduction in tumour density, was associated to long PFS, presumably due to intralesional necrosis.

Lyrdal *et al*. studied 10 patients with histologically proven metastatic RCC (overall 52 lesions) and evidence of disease progression despite previous cytokine treatment, who underwent PETCT using FDG at baseline and 1–2 months after sorafenib therapy.^19^ Post-therapy glycolytic activity and percentage decrease in glycolytic activity were measured on both soft tissue and skeletal lesions; lesion diameter was assessed by using diagnostic CT. In all lesions, mean FDG uptake significantly decreased to 75% compared to the initial values (71% in 39 soft tissue lesions and 82% in 13 skeletal ones). Best responders, with a percentage decrease greater than 20%, had significantly better mean OS than patients with least response (18.1 *vs.* 12.9 months); however, no significant correlation was observed between decrease in FDG uptake and PFS. A significant 20% decrease in soft tissue lesions diameter was observed on diagnostic CT. The authors demonstrate that PET-CT using FDG is a promising modality to evaluate response to sorafenib in both soft tissue and skeletal metastases of RCC; a clear advantage in comparison with RECIST evaluation was observed, since RECIST is limited to soft tissue lesions.

Minamimoto *et al*. analysed a prospective series of 12 patients with a total of 42 metastatic RCC lesions undergoing either sorafenib (7 patients) or sunitinib (5 patients) treatment; PET co-registered with contrast-enhanced CT (ceCT) was performed at baseline and after one cycle of therapy in all patients, to evaluate the early response and to predict PFS.^24^ According to EORTC 1999 criteria, patients were distinguished into metabolic partial response (SUVmax decreased > 25%), stable disease (SUVmax change less than 25%) and progressive disease (SUVmax increased > 25%).^28^ PET and CT were consistent in defining disease status in 8 patients, mostly with stable disease. Only a moderate reduction in mean SUVmax was noticeable in both sunitinib- and sorafenib-treated patients, with no statistically significant differences in PFS between the two therapy subsets. However, significant differences in PFS were confirmed between partial response and stable disease patients, as well as between partial response and progressive disease patients; besides, patients with PET-defined metabolic response showed longer PFS than patients with metabolic progressive disease.

Fourteen patients with metastatic RCC on-going first-line or second-line sunitinib treatment after cytokine and/or vascular endothelial growth factor (VEGF)-capture therapy failure were reviewed by Revheim *et al*.^25^ PET-CT using FDG was performed before and after two treatment periods in all but one patient with highly metabolic lung and nodal lesions, who showed rapid deterioration and poor prognosis before post-therapy PETCT examination could be obtained. The authors have found that patients with relatively low baseline FDG uptake in targeted lesions (SUVmax < 5) had significantly longer PFS than patients with relatively high baseline FDG uptake (SUVmax > 5); moreover, patients with SUVmax < 5 showed improved outcome after 3 months of follow-up, compared with patients with SUVmax > 5. Partial metabolic response according to EORTC criteria well correlated with longer PFS; Motzer scoring system (poor *vs.* intermediate) did not significantly correlate with either PFS or baseline FDG uptake.

The relevance of sequential PET-CT using FDG, performed at various intervals after therapy with sunitinib in patients with newly diagnosed meta-static RCC, as a surrogate marker of response to therapy was investigated by Kayani *et al*., as a part of a prospective phase II multicentre trial.^23^ Forty-four patients underwent PET-CT at baseline; 43 of them repeated this examination after the first cycle (at 4 weeks) and 39 had a third PET-CT scan after the third cycle of sunitinib (at 16 weeks). Changes in SUVmax between the baseline and 4-weeks, as well as between the baseline and 16-weeks, were calculated and compared with outcome (PFS and OS) data. The authors have demonstrated that both a SUVmax higher than 7.1 and a higher number of active lesions (more than 8) at baseline were predictive of shorter OS. Furthermore, despite evidence of metabolic response in 24/43 patients after one cycle of therapy and a median 22% reduction in SUVmax at the site of the previously most active lesion, there was no significant correlation between median reduction in SUVmax and PFS or OS, irrespective of the SUVmax at baseline. Similar results were obtained in 16/39 patients who showed metabolic response after the third cycle of therapy (16% reduction in SUVmax); however, a negative correlation was observed between disease progression and OS. Finally, a higher baseline SUVmax was negatively associated with metabolic response at both 4-weeks and 16-weeks scans (7.1 in metabolic non-responders; 4.4 in metabolic responders), whilst 10/12 patients with disease progression on 16-weeks PET-CT had been metabolic responders after one cycle of therapy (that is, 4-weeks scan had no prognostic significance).

A prospectively conducted study protocol by Ueno *et al*. on 30 histologically confirmed metastatic RCC patients undergoing sunitinib or sorafenib treatment was evaluated by using PET-CT before treatment and after 1 month of therapy.^20^ SUVmax of all lesions was calculated to obtain the mean SUVmax of the individual patient on both baseline and post-therapy examination; the mean ratio of SUVmax change and mean ratio in lesion diameter change on CT were obtained to classify patients as good, intermediate or poor responders, and compared with mean PFS and OS for each response group. Despite no complete response was obtained, an average reduction in mean SUVmax from baseline to post-therapy examination was observed (−18%; range −55 to 65%), and a slight reduction in mean lesion diameter (−6%; range −30 to 30%), with no significant differences across sub-types of tumour (clear cell *vs* papillary carcinoma). The Cox-analysis survival of good (lesion diameter sum not increased and SUVmax reduced > 20%), intermediate (lesion diameter sum not increased and SUVmax reduced < 20%) and poor responders (lesion diameter sum increased or appearance of new lesions) showed statistically significant difference in PFS as well as in OS. By using classical EORTC criteria for patient classification, instead, no association was observed between PFS and degree of response. Authors demonstrate that using a combination of PET (metabolic response) and CT (tumour size response) criteria in spite of classical EORTC criteria could predict PFS and OS in these patients.

Lastly, Khandani *et al*. have prospectively investigated eventual differences in the intensity of FDG uptake at baseline in clear cell and non-clear cell RCC, and whether changes in metabolic burden in targeted lesions could predict response to sorafenib in 17 patients.^18^ Therefore, PET-CT images were acquired at baseline and at completion of therapy, before nephrectomy was performed; baseline SUVmax and relative changes in SUVmax at post-therapy scan were calculated for each patient and tumour subtype. Clear cell RCC patients showed lower SUVmax at baseline than non-clear cell RCC (3.9 *vs.* 7.9); an inverse correlation was found between the metabolic activity of clear cell RCC primary tumour at baseline and the degree of size response to sorafenib on CT (correlation not found for non-clear cell RCC). Due to the limited sample size (13 clear cell and 4 non-clear cell RCC), only a weak inverse correlation was detected between relative change in SUVmax and tumour size response, suggesting a limited relationship between metabolic effects of sorafenib and morphological changes on CT. Finally, no significant differences in the rate of recurrence and outcome measures were found between patients with high baseline SUVmax (> 4) and low baseline SUVmax (< 4).

## Discussion

It is well known that FDG is physiologically excreted by the urinary system, therefore hampering the accurate assessment of the primary renal lesion in terms of metabolic burden and aggressiveness, as well as interfering with the evaluation of response after targeted therapy. However, in recent years, PET-CT using FDG has played an increasingly important role in the management of patients affected by primary renal cell malignancies, specifically for the evaluation of metastatic lesions. In fact, as reported by Wang *et al*. in a recently published meta-analysis on fourteen studies, PET-CT using FDG is a reliable diagnostic tool for the detection of extra-renal lesions of RCC, with pooled sensitivity and specificity values of 79% and 90%, respectively (in the same studies, the authors report a pooled sensitivity and specificity of 62% and 88%, respectively, for renal lesions).^16^

Traditional therapeutic regimens with cytotoxic agents and/or radiation therapy fail in most patients with advanced RCC, with a significant toxicity burden; neither interleukin-2 nor interferon alpha, alone or in association with cytotoxic drugs, show evidence of long-term efficacy in this setting, with no significant benefit on the survival and recurrence rate.^29^–^32^ Therapy with TKIs, alone or in combination with immune-chemotherapeutic agents is currently performed in patients with metastatic RCC, with satisfactory results.^10^–^13^ A recent Swedish register-based study has demonstrated a significant impact of the duration of first-line treatment when sorafenib is used in sequential therapy with sunitinib in patients with RCC.^33^ Eichelberg *et al*. have shown that 50% patients with metastatic RCC, previously undergoing sorafenib with unsatisfactory results, benefit from a secondary use of sunitinib, with a significant increase in progression-free survival from 8–10 to 17 months.^34^

As demonstrated by our review, in the last three years PET-CT using FDG has been increasingly performed to assess the therapeutic efficacy of TKIs (notably, sunitinib and sorafenib) in patients with metastatic RCC, irrespective of previous treatments or nephrectomy. Reduction in FDG uptake from pre-therapy to post-therapy evaluation is used by all selected studies as an estimate of treatment response: therefore, a baseline PET-CT examination showing significant metabolic activity within lesions is mandatory to correctly assess the response to therapy at the post-treatment scan. Within a prospective cohort of patients by Kayani *et al*., 43 patients from a total of 44 have undergone PET-CT after 1 cycle of sunitinib (4 weeks), since the remaining patient had a negative baseline scan.^23^ Likewise, in the same study, 39 patients were reevaluated after 3 cycles of sunitinib (16 weeks): however, the lesion-based analysis of response had to exclude previous pathologic sites which showed a complete/near complete normalization of FDG uptake after 1 cycle of therapy.

In all the selected studies outcome measures such as PFS and OS were included: authors compared them among groups of patients derived in accordance to the disease status as assessed by post-treatment PET-CT. A slight-to-moderate reduction in FDG uptake from baseline to post-therapy scan was observed in most patients, while only a minority of patients showed disease progression; a complete metabolic response (*i.e*. complete normalization of FDG uptake in all lesions in a single patient) was never achievable. Actually, in many studies, a good correlation was found between partial metabolic response and PFS or OS, with the highest survival rates in patients showing the greatest reduction in SUVmax.^19^,^20^,^24^,^25^ Thus, PET-CT appears to show a high predictive value in the evaluation of response to therapy in both skeletal and soft tissue metastases of RCC. However, contradictory results arise from our literature analysis. In their pilot study, Vercellino *et al*. also have observed a longer PFS in patients with partial metabolic response rather than in patients with stable disease or progression; however, a statistical significance was not reached, presumably due to their small sample size (12 patients).^22^ Similarly, Khandani *et al*. detected only a weak inverse correlation between relative change in SUVmax and tumour size response, and no correlation with PFS or OS, in 13 patients with metastatic clear cell RCC.^18^ On a larger population, Kayani *et al*. have found no correlation between median reduction in SUVmax and PFS or OS, especially when PET-CT was performed after 1 cycle of therapy; as authors themselves state, the exclusion of patients with clinical or radiological progression from the sequential PET-CT analysis could have influenced these results somehow.^23^ Conversely, they found that higher SUVmax at baseline and higher number of lesions were predictors of shorter OS, as observed in other studies.^18^,^25^

Increase in FDG uptake (*i.e*. a metabolic progression of disease) was associated with lower OS, though not always with a less favourable PFS.^23^

Some studies have compared the predictive value of post-therapy PET-CT using FDG and clinical/morphological criteria and scores.^19^,^20^,^24^,^25^ In these settings, post-therapy PET-CT performed better than clinical scores (Motzer score) or morphological criteria (RECIST) in predicting PFS and OS, therefore resulting in a stronger prediction of response to treatment. As observed by Lyrdal *et al*., post-therapy PET-CT is more useful than RECIST criteria, particularly for the evaluation of skeletal lesions, as RECIST is limited to soft tissue lesions.^19^ RECIST criteria exhibit significant limitations when response to cytostatic (like TKIs), rather than cytotoxic therapies (traditional chemotherapeutic agents, interferon), has to be evaluated, therefore emphasizing the role of metabolic changes on post-treatment PET-CT in this setting. Furthermore, using a combination of metabolic activity assessment (lowering in FDG uptake) and morphological changes (reduction in tumour size) better contributes to predict PFS and OS, rather than metabolic assessment alone.^20^ Reduction in lesion size is not an accurate predictor of good response, by itself: responding lesions sometimes showed an increase in size, despite extensive necrosis, evidence of low FDG uptake and high PFS.^22^

Most authors have used SUVmax, which is an indirect estimate of the glycolytic activity in the most active pixel within the lesion, as an index to detect eventual changes in metabolic activity from baseline to post-treatment scan. The absolute (SUVdiff) or relative (SUVrel) variation in SUVmax have been used as they reflect the changes in the amount of vital tumour cells induced by therapy. However, active inflammation in sites of responding lesions could also accumulate FDG; this could partly explain the apparent low correlation with PFS and OS observed in some studies.

Some studies showed a negative correlation between baseline SUVmax in the most active lesion and outcome.^18^,^23^,^25^ Higher baseline SUVmax and higher number of metabolically active lesions were significantly associated with greater risk of disease progression and poorer PFS or OS. Moreover, patients with higher baseline SUVmax showed a lower response rate than patients with lower baseline SUVmax, even after one cycle of therapy.^23^

Interestingly, the efficacy of PET-CT in the prediction of response to treatment and patient outcome was not affected neither by the therapeutic drug used (sorafenib *vs.* sunitinib), nor by the histological subtype of tumour; only a slight difference in baseline SUVmax was observed, with lower mean values in clear cell RCC. Khandani *et al*. have found that changes in SUVmax weakly correlated with tumour size response only in clear cell RCC patients, while non-clear cell RCC did not; however, only 4 non-clear cell RCC patients were included, therefore limiting the statistical relevance of the results.^18^

## Conclusions

The role of PET-CT using FDG in assessing the response to TKIs in metastatic RCC patients is still not well defined, partly due to heterogeneity of available studies. However, PET-CT reveals potential role for the selection of patients undergoing therapy with TKIs. The use of contrast-enhanced PET-CT appears to be promising for a “multi-dimensional” evaluation of treatment response in these patients.

## Figures and Tables

**TABLE 1.** Basic study characteristics

**Authors**	**Journal/year**	**Country**	**Study design**	**Patients performing PET-CT**	**Mean age**	**%Male**
Vercellino *et al*.^22^	Cancer Biother Rad 2009	France	Prospective	12	59	83
Lyrdal *et al*.^19^	Nucl Med Commun 2009	Sweden	Prospective	10	61	80
Minamimoto *et al*.^24^	Clin Nucl Med 2010	Japan	Prospective	12	61.5	67
Revheim *et al*.^25^	Clin Oncol 2011	Norway	Prospective	14	60	*NR*
Kayani *et al*.^23^	Clin Cancer Res 2011	UK	Prospective	44	61	75
Ueno *et al*.^20^	BMC Cancer 2012	Japan	Prospective	30	64	83
Khandani *et al*.^18^	Nucl Med Commun 2012	USA	Prospective	26	59.5	73

NR = Not reported

**TABLE 2.** Treatment and post-therapy PET/CT evaluation

**Authors**	**Therapy (n)**	**Prior nephrectomy (%)**	**Patients performing post-therapy PET-CT scan**	**Post-therapy PET scan timing**
Vercellino *et al*.^22^	Sunitinib (12)	10 (83)	12	1 cycle
Lyrdal *et al*.^19^	Sorafenib (10)	9 (90)	10	1 cycle
Minamimoto *et al*.^24^	Sunitinib (5) Sorafenib (7)	10 (83)	12	1 cycle
Revheim *et al*.^25^	Sunitinib (14)	13 (93)	13^*^	2 cycles
Kayani *et al*.^23^	Sunitinib (44)	No	43^**^	1 cycle (43 pts) 3 cycles (39 pts)
Ueno *et al*.^20^	Sunitinib (16) Sorafenib (14)	22 (73)	30	1 cycle
Khandani *et al*.^18^	Sorafenib (26)	No	17	1 cycle

* Not performed in 1 patient because of rapid status deterioration

** Not performed in 1 patient because of negative baseline scan

**TABLE 3.** FDG uptake quantification indexes and response

**Authors**	**Quantification indexes**	**Partial response**	**Stable disease**	**Disease progression**
Vercellino *et al*.^22^	SUVmax %SUVmax	4	7	1
Lyrdal *et al*.^19^	SUVmax SUVmean	*NR*	1	8
Minamimoto *et al*.^24^	SUVmax %SUVmax	12	6	3
Revheim *et al*.^25^	SUVmax	6	3	4
Kayani *et al*.^23^	SUVmax %SUVmax	24	14	4
Ueno *et al*.^20^	SUVmax	12	10	8
Khandani *et al*.^18^	SUVmax %SUVmax	*NR*	*NR*	*NR*

NR = Not reported
